# Spontaneous mind wandering impairs model-based decision making

**DOI:** 10.1371/journal.pone.0279532

**Published:** 2023-01-26

**Authors:** Shuyan Liu, Milena Rabovsky, Daniel J. Schad

**Affiliations:** 1 Department of Psychiatry and Psychotherapy, Charité – Universitätsmedizin Berlin, Berlin, Germany; 2 Department of Psychology, University of Potsdam, Potsdam, Germany; 3 Psychology Department, Health and Medical University, Potsdam, Germany; Children’s Hospital of Los Angeles, UNITED STATES

## Abstract

**Background:**

If our attention wanders to other thoughts while making a decision, then the decision might not be directed towards future goals, reflecting a lack of model-based decision making, but may instead be driven by habits, reflecting model-free decision making. Here we aimed to investigate if and how model-based versus model-free decision making is reduced by trait spontaneous mind wandering.

**Methods and findings:**

We used a sequential two-step Markov decision task and a self-report questionnaire assessing trait spontaneous and deliberate mind wandering propensity, to investigate how trait mind wandering relates to model-free as well as model-based decisions. We estimated parameters of a computational neurocognitive dual-control model of decision making. Analyzing estimated model parameters, we found that trait spontaneous mind wandering was related to impaired model-based decisions, while model-free choice stayed unaffected.

**Conclusions:**

Our findings suggest trait spontaneous mind wandering is associated with impaired model-based decision making, and it may reflect model-based offline replay for other tasks (e.g., real-life goals) outside the current lab situation.

## Introduction

When making decisions, we often think about the consequences of the decision to guide goal-directed choices, but we also rely on habits reflecting the past. During mind wandering, our thoughts are distracted from the current external task (e.g., of making a decision), and are instead focused on other thoughts. We here were interested in whether such mind wandering impairs the goal-directed aspects of decisions, while leaving the action of habits unaffected.

We often base our decisions on two systems [1]: habits (model-free) and goals (model-based). Computational models have described habits as depending on model-free retrospective reinforcement learning that simply repeats the actions that lead to a gain in the past, and goals as relying on model-based prospective planning that is guided by the likelihood of affective outcomes that are predicted by a model of the environment [2,3]. Habits and goals work in tandem and each system has an equally valuable approach to guiding action selection [4]. Adaptive behavior depends on the ability to flexibly regulate their respective contributions under varying contexts [3,4].

Theoretically [2,3], model-based decision-making is thought to rely on cognitive computations that involve anticipating the consequences of one’s actions based on a cognitive model of the environment. Consistent with this theory, previous studies showed that model-based decision making is impaired under dual-task load [5] and during transcranial magnetic stimulation of the dorsolateral prefrontal cortex [6], and depends on cognitive capacities such as working memory and processing speed [7,8].

Here we ask whether our ongoing decision making process is also disrupted by internal information, for example, in the form of unintentional and intentional task-unrelated imagery and thoughts (i.e., mind wandering) that are unhelpful to the task at hand [9–11]. Patterns of ongoing thought vary across different tasks and individuals [12] and dynamically change across time, such that an individual may have multiple spontaneous alternations between task focus and off-task thoughts [13].

Mind wandering reflects the capacity to disengage internal thoughts from the external environment, known as perceptual decoupling [14]. According to the decoupling hypothesis mind wandering is associated with a reduction in cognitive processing of external environment, such that it can be devoted to internal thoughts [14,15]. This is hypothesized to result from executive control, which shields internal thought against external distractions [10,16,17]. Model-based decision making involves learning and reasoning about the external environment [2,3]. In situations of mind wandering cognitive capacities such as working memory [18–20] needed to support model-based decision making may not be available to the external decision-task. Instead of processing task-related information (i.e., learning about the future consequences of potential choices), the model-based decision making may process internal task-unrelated information related to mind wandering.

Perceptual decoupling occurs not only in an all-or-none fashion, where people are either mind wandering or focused on a given task, but also happens in a graded fashion, reflecting states of weak and deep mind wandering, known as the levels of inattention hypothesis [21]. Weak mind wandering is thought to impair high-level cognitive processing, but to leave low-level processing intact [21]. In the current context, this may be taken to suggest that weak mind wandering may reduce model-based decision making that requires more complex and higher-level cognitive processing as compared to an unaffected model-free decision system. In individuals reporting high levels of mind wandering, one would, therefore, be expected to find impairments of model-based decisions, but not of model-free decisions.

One distinction in mind wandering research is whether mind wandering occurs spontaneously (i.e., uncontrolled, unintentional) or deliberately (controlled, intentional) [22]. Deliberate mind wandering may occur in situations where external demands for cognitive processing are absent or are intentionally ignored [9,23]. However, during cognitively demanding lab experiments, subjects are motivated to perform well on the task and may not engage in deliberate mind wandering [24]. By contrast, spontaneous mind wandering is not under deliberate cognitive control and will therefore occur also during a demanding cognitive lab task [24]. In line with this view, perceptual decoupling seemed to be more closely associated with spontaneous than deliberate mind wandering [25]. Marcusson-Clavertz and Kjell [26] showed that spontaneous mind wandering was strongly positively associated with poor attentional control.

The standard for assessing model-free and model-based choice control is the two-step Markov decision task [3]. In this task, we expected that spontaneous mind wandering will interfere with model-based decision-making, because model-based decision-making relies on cognitive resources such as working memory [2,7,8]. When subjects use cognitive resources to process internal information (i.e., during mind wandering), cognitive resources for external information (i.e., decisions) will be reduced [10,17]. Moreover, we did not expect subjects to engage in deliberate mind-wandering in the two-step task, which is a highly dynamic decision-making task. This is due to task difficulty. The two-step task does not involve only a simple choice in a single goal but a multistep decision. At the first step, participants choose between two options that determine the presentation of the second-step choices. The reward probabilities associated with the second-step choices slowly and randomly change across trials, which requires continuous learning. Thus, subjects need to constantly engage in learning since the optimal actions are continuously changing across trials. The literature shows that individuals report more spontaneous mind wandering on difficult tasks and more deliberate mind wandering on easy tasks [24]. Moreover, participants are more likely to control their deliberate mind wandering when they are aware of task difficulty, which is the case in the two-step task due to detailed task instructions. A study by Robison & Unsworth (2018) showed that cognitive and contextual factors can have distinct relationships with spontaneous and deliberate mind-wandering [23]. Last, when subjects hit top 3 on the task performance, they were expecting to receive a surprise gift (i.e., a box of chocolates). We thus expected that subjects would be motivated to not engage in mind wandering deliberately, but to deliberately focus on the task to receive a surprise reward. Therefore, we expected that spontaneous mind wandering would interfere with model-based decision making, whereas our measure of deliberate mind wandering would not relate to model-based decision-making in the two-step task because subjects would not engage in deliberate mind wandering.

We here tested our hypotheses by measuring trait spontaneous and deliberate mind wandering [22] together with the well-established two-step Markov decision task [3]. We hypothesized that trait spontaneous but deliberate not mind wandering would be associated with impaired model-based but not model-free decision making.

## Methods

### Subjects

Thirty-three right-handed healthy subjects participated in our study after giving informed consent (17 females; mean age: 24.6 years, *SD* = 3.5). The sample size was estimated in reference to a previous study on cognitive abilities in decision-making, which found a strong effect of individual differences in cognitive processing speed on model-based choice [7]. We performed a formal power analysis. We expected that mind wandering would interfere with cognitive model-based processing. This is known to relate closely to individual-difference measures of cognitive processing speed [7]. We therefore assumed that spontaneous mind wandering would have the same standardized effect size as a previous finding on cognitive processing speed [7]. To compute power, we z-standardized cognitive processing speed and estimated its influence on model-based control using linear regression based on the prior data set [7]. This yielded an estimate (regression coefficient) of *b* = 0.240. We used this effect estimate (i.e., *b* = -0.240; since we expected high mind wandering to reduce model-based control) for the power analysis, which was also based on linear regression analysis. First, we used two-tailed tests and an alpha level of 0.05, and we found a power of 65%. Second, based on our a priori directed hypothesis that mind wandering impaired model-based decision-making, we performed another otherwise equivalent power analysis using one-tailed tests (also with an alpha level of 0.05). With this, the power was 76%, and thus of reasonable magnitude.

Spontaneous and deliberate mind wandering were measured via the two four-item Mind Wandering Spontaneous (MW-S) and Mind Wandering Deliberate (MW-D) self-report scales [22]. Mind wandering data was available for thirty-two subjects and one subject with missing mind wandering data was excluded from analysis. Subjects moreover performed the digit symbol substitution test (DSST; [27], which measures cognitive processing speed and is a sub-component of fluid intelligence [7]). Subjects were compensated a fixed amount. Moreover, when subjects hit top 3 on the task performance, they were expecting to receive a surprise gift (i.e., a box of chocolates). The study was approved by the Ethics Committee at Charité –Universitätsmedizin Berlin and was conducted in accordance with the principles of the 1964 Declaration of Helsinki.

### Procedure

**Fig 1:**
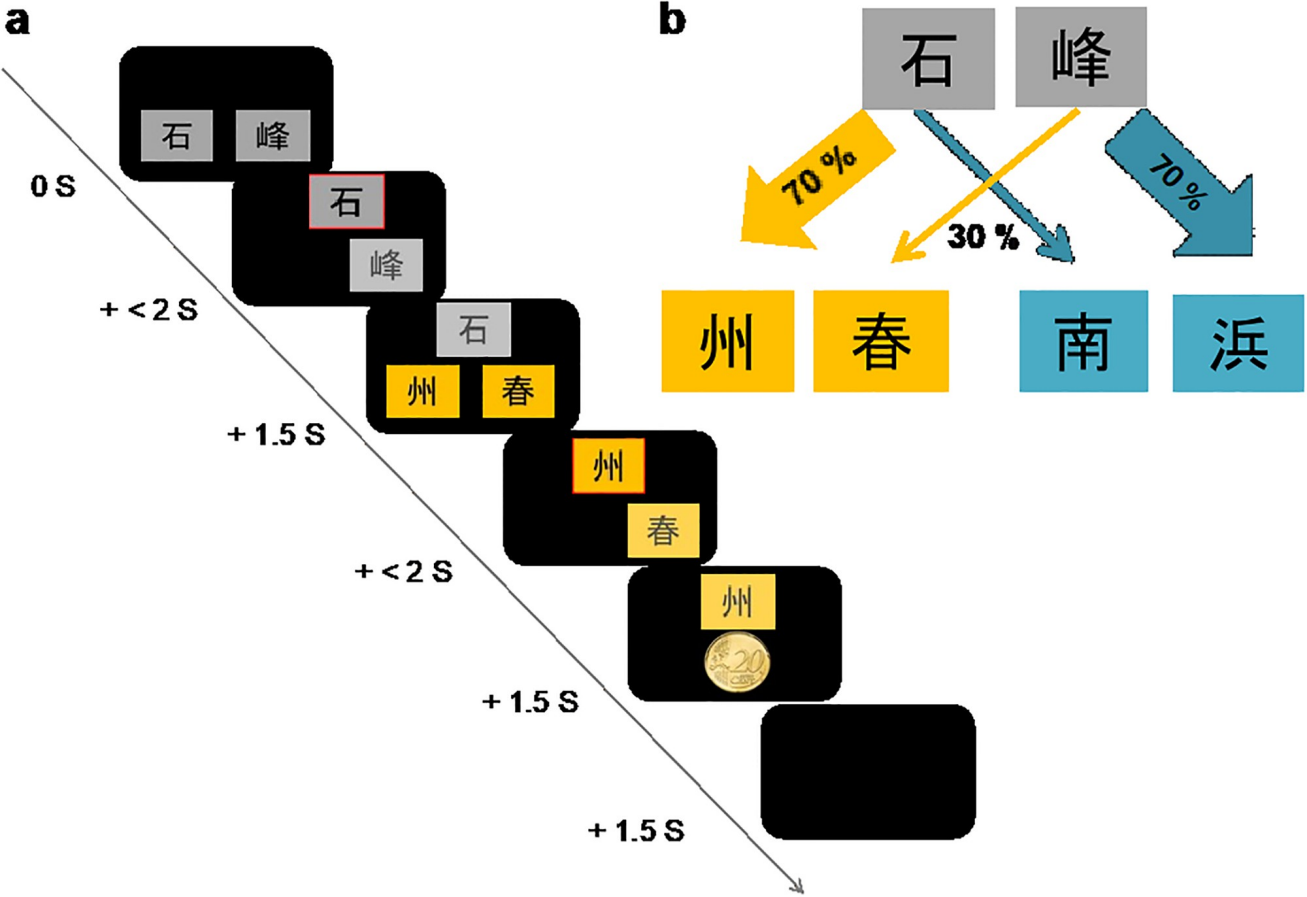
Two-step decision task. (a) Trial structure. Each trial consisted of choices at two steps. Step 1 involved the first choice between two abstract gray stimuli. The chosen stimulus was framed with red color in the center-top of the screen for 1.5s. Subsequently, subjects were presented with another stimulus pair in step 2. The second choice was either rewarded with money (20 cents) or followed by no win. (b) The transition probabilities from step 1 to step 2 remained fixed, reflecting either common transitions (70% of trials) or rare transitions (30%). The reward probabilities for each stimulus in step 2 changed independently based on Gaussian random walks with reflecting boundaries at 25% and 75%. Win probabilities varied, therefore, as a function of the trial number.

Subjects performed the two-step Markov decision task by [3], which is designed to measure model-based and model-free control in decision making (see Fig 1). The task consists of two subsequent steps, each demanding a choice between two stimuli. During the first step (step 1), two stimuli are presented, and subjects are requested to choose one. This choice probabilistically determines (i.e., a common (70%) or a rare (30%) transition) which pair of stimuli are presented at the second step. During the second step (step 2), subjects are presented with another pair of stimuli and again are requested to choose one. This second choice is either rewarded with money (here: 20 cents) or not rewarded. Prior to the experiment, subjects are instructed that the reward probabilities in step 2 slowly change probabilistically over trials, but that the probabilities controlling the transitions from step 1 to step 2 remain fixed and are primarily associated with one or the other of the second-step states.

The model-free and the model-based system make distinct predictions for task performance. The model-free system only knows about rewards but does not know about transition probabilities. Therefore, the model-free system predicts that rewarded first-stage actions are repeated in the next trial, whereas unrewarded actions are not repeated. The model-based system, but contrast, knows about rewards and about transitions, and predicts an interaction between reward and transition probability. For example, if a rare transition (30%) leads to a reward, it is better not to repeat this action, but to switch to the other first-stage choice in the next trial. Learning of the two systems is best captured by computational reinforcement learning modeling, which provides parameter estimates for each subject of how strongly each system determines choices in each subject.

To familiarize with the structure of the task, subjects underwent 50 practice trials with a separate set of stimuli. The main task consisted of 201 trials, in three blocks of 67, separated by breaks [3]. In the present study, subjects performed the main task twice, interspersed with short breaks, where subjects performed either video gaming or were listening to music (order counterbalanced). Results for these break scenarios are reported elsewhere [28]. Mind-wandering questionnaires were collected after the two-step task.

### Analysis

In our analysis approach, we aimed to test our specific theoretical hypotheses in one consistent computational analysis, taking account of the full data at once. To this end, we used a computational dual-control model by [3], and used the re-parametrization introduced by [8]. The model suggests reward-based choice to originate from two distinct systems of model-free versus model-based reinforcement learning (RL). Model-free RL is implemented as SARSA (*λ*) temporal difference (TD) learning [29] and model-based RL implements Bellman’s equation [30] assuming that expected maximal outcomes at the second stage are rationally weighted by their (transition) probabilities, which are taken to be known and fixed [3]. Throughout the task, both algorithms thus learn expected (Q-) action values. For a full model description see the S1 File (also the supplement in [3]).

The model contains seven free parameters: (*i+ii*) separate weights for the model–based system, *β*_*MB*_, and for the model-free system, *β*_*MF*_; (*iii*) *β*_*2*_, the inverse temperature parameter at the second-step controls how deterministic choices are at the second decision-stage and is thought to exhibit contributions from model-based and model-free control; (*iv+v*) *α*, learning rate of the model-free system for the first- (*α*_*1*_) and second-step (*α*_*2*_); (*vi*) *λ*, reinforcement eligibility parameter of the model-free system as the relative degree of second-step reward prediction errors to update first-step model-free values; (*vii*) *p*, first-step choice perseveration, which is neither associated with model-free nor with model-based control.

Some of the model parameters are constrained to certain ranges. E.g., learning rates are bounded between 0 and 1, and weighting parameters are bounded to be larger than zero. For bounded parameter estimation and due to the normal distribution assumption in parameter estimation and statistical testing, we transformed bounded model parameters to an unbounded scale via a logistic transformation [x’ = log(x/(1-x))] for parameters α and λ (*α*_*1*_, *α*_*2*_ and *λ*), and via an exponential transformation [x’ = exp(x)] for parameters β (*β*_*MB*_, *β*_*MF*_ and *β*_*2*_).

We estimated model parameters for each subject using expectation maximization (EM; as described in [7,31]) using custom computer code. The estimated model parameters were taken from our prior work [8,28] and were not re-estimated. To model the two sessions of 2-step, we estimated (1) one parameter capturing the mean parameter estimate across both sessions, and (2) one parameter estimating the difference in the estimate between sessions, yielding 7 (model parameters) x 2 (mean + difference) = 14 model parameters, which were simultaneously estimated using EM. For analysis, we discarded the parameters capturing the difference between sessions, because we were not interested in session differences, but focused our analysis on the mean estimates for each of the 7 model parameters.

To validate the inference procedure, we performed parameter recovery. For this, we treated the estimated model parameters per subject as the “true” parameters, and simulated behavioral data based on these true parameters. Next, we estimated parameters from the simulated data (“recovery parameters”) and compared “true parameters” to the “recovery parameters”. Correlations between true and recovery parameters were generally high (*r* > = 0.8; see S1 Fig). In particular, for the critical model-based weight, the correlation was at *r* = 0.88, reflecting very good recovery.

Next, we studied covariance among estimated model parameters. Indeed, some of the model parameter estimates were highly correlated (see S2 Fig). Specifically, the 2^nd^-stage weight was correlated with the model-based weight, which may reflect the fact that model-based control also contributes to choices at the 2^nd^-stage. Moreover, the model-based weight was also highly correlated with the choice repetition parameter, possibly because both reflect a low degree of randomness of choices.

We used linear models (for between-subject analyses) and linear mixed-effects models (for analyses comprising repeated measures) to test the influence of mind wandering on two-step model parameters. First, we performed each analysis using continuous mind wandering scores. To investigate the robustness of our results, we also used a median-split and compared subjects who reported a low versus a high level of mind wandering. Moreover, we analyzed the influence of continuous mind wandering scores on model-free and model-based weights by performing null hypothesis Bayes factor analyses [32]. We reasoned that the effect of mind wandering should be similar to the a priori known influence of cognitive abilities (i.e., working memory and processing speed; [5,7,8] on model-based decision-making. The strongest and most robust effect in our prior analyses [7] showed that model-based choice was enhanced in individuals with high processing speed as assessed in the digit symbol substitution test (DSST; [27], and we used this influence of DSST on computational model-based parameters (i.e., the likelihood; M = -0.24, SD = 0.11) to define the prior for the regression coefficient of spontaneous mind wandering. Moreover, we used a linear mixed-effects model to test the directed hypothesis (one-tailed) that the influence of spontaneous mind wandering on model-based choice is stronger than on model-free choice. In this analysis, to investigate whether the influence of spontaneous mind wandering on behavioral control was stronger for the model-based than the model-free weight, we coded the estimated parameter value as a within-subject factor “parameter” (with two levels: model-free weight and model-based weight). We used a linear mixed-effects model to predict the estimated parameter value based on spontaneous mind wandering, the within-subject factor “parameter”, and their interaction. The interaction term of this analysis was taken to test whether the influence of spontaneous mind wandering was stronger for the model-based compared the model-free weight. Again, we performed a null hypothesis Bayes factor analysis, where the prior for the interaction (model parameter [model-free vs. model-based] x spontaneous mind wandering) was set to a normal distribution with mean -0.24 (SE = 0.11).

We also performed analyses for the other model parameters, again using frequentist and null hypothesis Bayes factor tests. As a prior, we used the influence of z-scaled DSST on z-scaled model parameters estimated in our prior work [7]: as the prior mean (standard deviation) of the normal prior distribution we used the mean (standard deviation) of standardized regression coefficient across model parameters, i.e., M = 0.32 (SD = 0.28). All analyses were performed using R software (R Foundation for Statistical Computing, Vienna, Austria). Differences were considered significant at *p* < .05.

## Results

We first performed consistency checks of whether the tasks were conducted appropriately. We found that self-reported spontaneous mind wandering had an average value of 4.11 (SD = 1.11) and deliberate mind wandering had an average value of 4.49 (SD = 1.06). However, these self-report scores did not correlate with average response times at the first or second step in the 2-step task (-0.106 < *r* < -0.038, *p*-values > .5), providing no separate validation for these self-report measures.

Concerning the 2-step task, we found that first-stage choices were correct (i.e., choosing the higher-valued option) on 50.2% (SD = 3.1; chance level = 50%) of trials, in line with prior results [33]. Importantly, this again shows that the 2-step task is too difficult for subjects to successfully select the correct first-stage actions, which is not surprising given the probabilistic feedback on current reward probabilities, and given that on each trial, feedback is provided on only one out of four terminal states. Importantly, however, strong and pervasive effects of “reward” and the “reward x transition interaction” on 1^st^-stage choice repetition evidence the effective use of model-free and model-based strategies in trying to solve the task. To study the quality of the computational model fit, we computed the average probability of the first-stage action based on the model, which was good with a value of 0.69 (SD = 0.11). In the computational model, the 2^nd^-stage learning rate was estimated as 0.59 (SD = 0.20), suggesting that subjects successfully learned the contingencies and updated their second-stage choices to obtain more reward. To study model fit more closely, and to compare model behavior to human behavior, we simulated data based on the estimated model parameters. S3 Fig shows the main effect of reward, reflecting model-free control, as well as the interaction of reward x transition probability, reflecting model-based control. The results (see S1 File) suggest that the model captured the key difference in model-based control between high versus low spontaneous mind wandering well, and also didn’t show a relation of spontaneous mind wandering to model-free learning, in line with the empirical findings.

**Fig 2:**
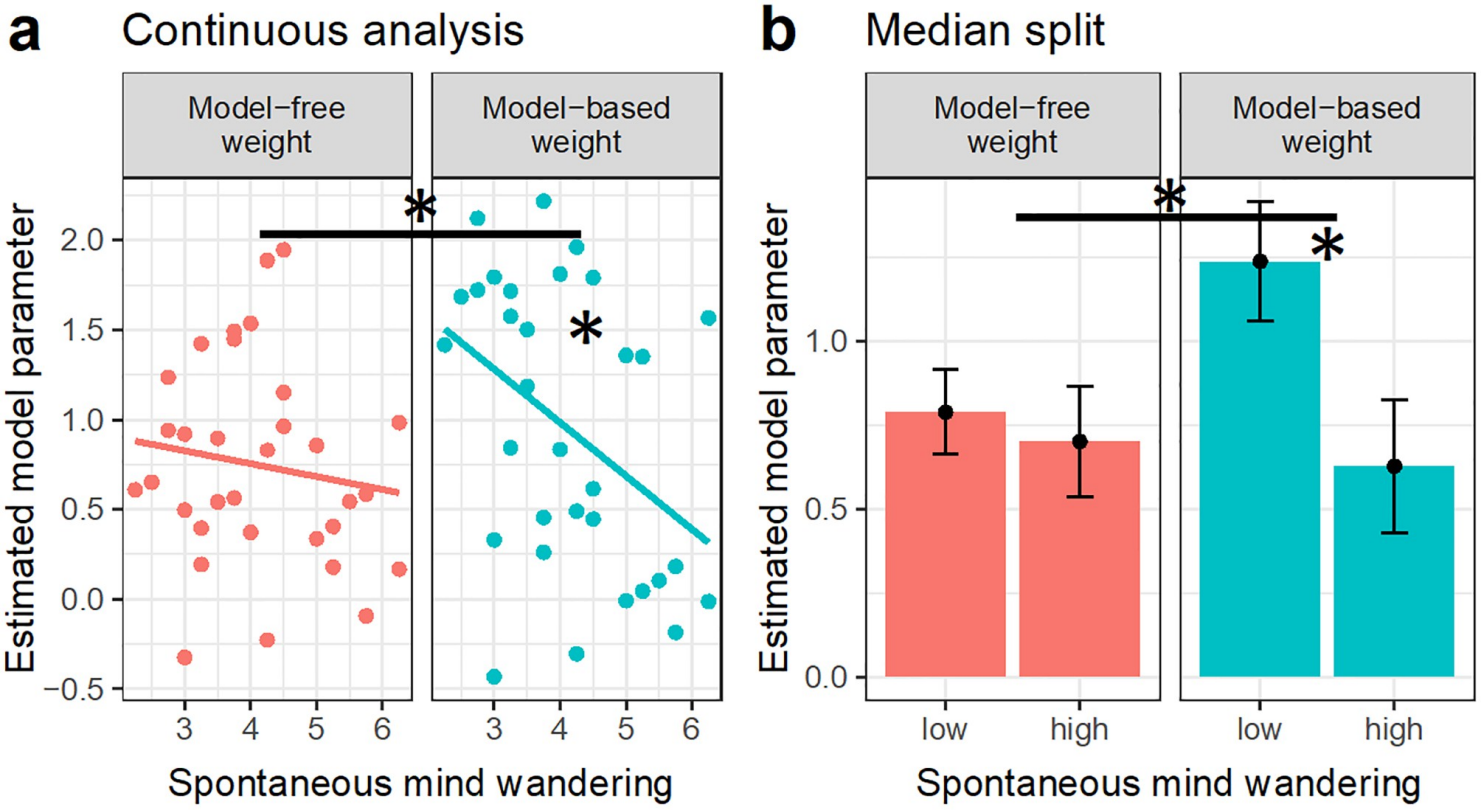
Spontaneous mind wandering and separate computational weights for the model-free system *β*_*MF*_ (red, left) and the model–based system *β*_*MB*_ (green, right) in (a) continuous analyses and (b) median-split analyses. Each dot indicates an individual subject’s data (*N* = 32). A median split was used for comparing subjects who reported a low versus a high level of spontaneous mind wandering. Error bars denote standard errors of the means (±SEM). Asterisks indicate significant linear effects/differences (*p* < .05). The influence of continuous spontaneous mind wandering on the model-based weight is moreover supported by a Bayes factor analysis (*BF*_*10*_ = 11.6).

We next tested our main hypothesis that high levels of spontaneous mind wandering are associated with reduced model-based control. Indeed, as is displayed in Fig 2, the model-based weight was reduced in individuals with high levels of spontaneous mind wandering (*b* = -0.30, *SE* = 0.12, *t* = -2.46, *p* = 0.020). This effect was also significant when comparing high versus low spontaneous mind wandering using a median-split (*b* = -0.61, *SE* = 0.27, *t* = -2.29, *p* = 0.029). Moreover, a Bayes factor null hypothesis analysis showed a Bayes factor of *BF*_*10*_ = 11.6 in favor of the H1, providing “strong” evidence [34] for an influence of spontaneous mind wandering on model-based control. We further validated the null hypothesis Bayes factor using a sensitivity analysis. The results (see S4 Fig) further supported our result that high levels of spontaneous mind wandering were associated with low levels of the model-based weight.

Next, we tested our second hypothesis that model-free control is not influenced by spontaneous mind wandering. Consistent with expectations, we found no evidence that spontaneous mind wandering influenced the model-free weight (continuous score: *b* = -0.072, *SE* = 0.093, *t* = -0.77, *p* = 0.44; median split: *b* = -0.087, *SE* = 0.20, *t* = -0.43, *p* = 0.67), in line with the view that model-free learning is independent of spontaneous mind wandering. However, a null hypothesis Bayes factor analysis revealed a Bayes factor of *BF*_*10*_ = 0.53 in favor of the H1 (i.e., *BF*_*01*_ = 1.88), reflecting only "anecdotal” evidence for the H0 [34].

We further tested whether the influence of spontaneous mind wandering on behavioral control was stronger for the model-based than the model-free weight, and indeed found this to be the case for the continuous mind wandering score (*b* = -0.23, *SE* = 0.13, *t* = -1.75, *p*_1-tailed_ = 0.045, *BF*_*10*_ = 0.55) and for the median-split comparison (*b* = 0.52, *SE* = 0.28, *t* = 1.87, *p*_1-tailed_ = 0.035, *BF*_*10*_ = 3.32); however, Bayesian evidence for this difference was only anecdotal to weak.

Next, we asked how the influence of spontaneous mind wandering on model-based decision-making compared to a very strong known influence on model-based choice, which is the influence of processing speed (i.e., DSST). We z-transformed predictors for comparison and found that in our present sample, the DSST showed the known strong influence on the model-based weight (standardized regression coefficient *β* = 0.374, *SE* = 0.130, *t* = 2.88, *p* = 0.007), and that the influence of spontaneous mind wandering was nearly as strong (*β* = 0.301, *SE* = 0.132, *t* = 2.28, *p* = 0.030). Statistically, we aimed to test whether the association of trait spontaneous mind wandering with the model-based weight differed from the association with the digit substitution task. To this end, we coded the model-based weight from each subject twice, once paired with the z-scaled trait spontaneous mind wandering score, and once paired with the z-scaled digit substitution task score. The trait spontaneous mind wandering scores and the digit substitution task scores were thus coded as one repeated-measures variable (“score”), and we coded a second repeated-measures factor (“test”), with the two levels “spontaneous mind wandering” and “digit substitution task”. We then estimated a linear mixed-effects model with the “score” variable as the dependent variance, the fixed-effects predictors “model-based weight”, the factor “test” (effect coded: -0.5, +0.5), and their interaction, as well as random intercepts for subjects. The results of this analysis showed no significant interaction of “score” and the model-based weight (*b* = -0.07, *SE* = 0.27, *t =* -0.26, *p* = .800), suggesting the association with the model-based weight did not differ between trait spontaneous mind wandering and the digit substitution task.

**Fig 3:**
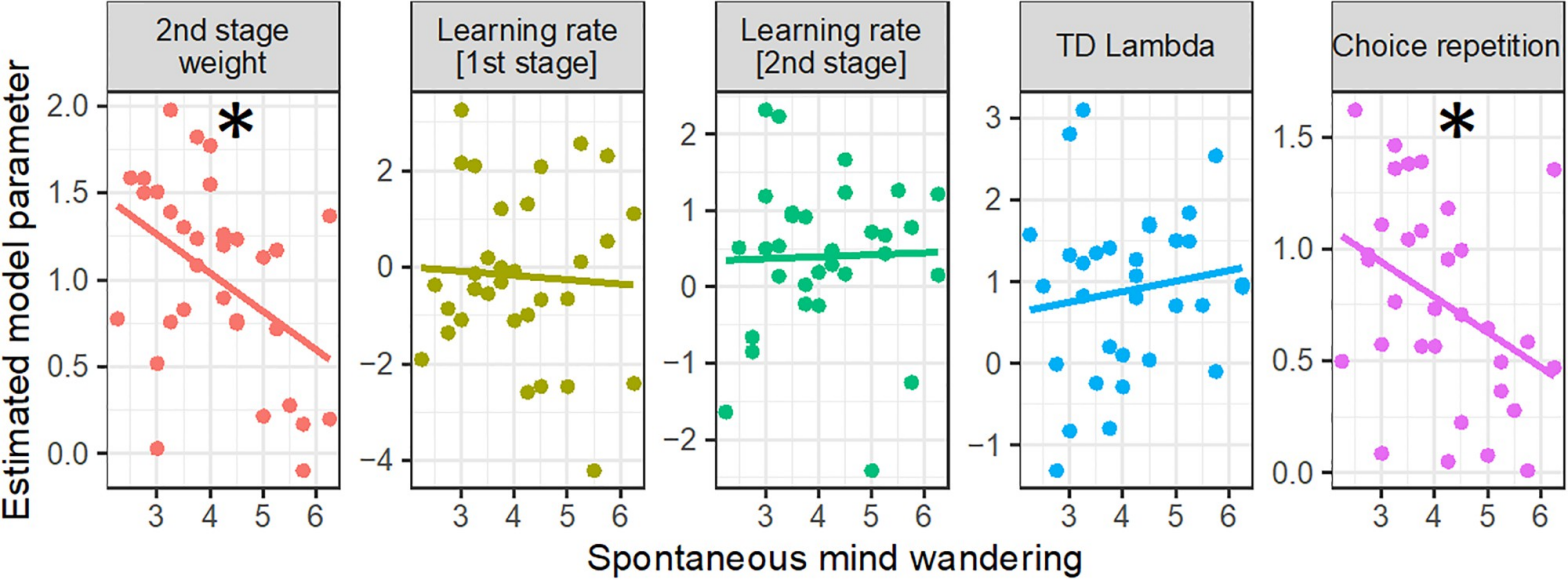
Spontaneous mind wandering and computational model parameters. Each dot indicates an individual subject’s data (*N* = 32). Asterisks indicate significant linear effects (*p* < .05); results are also supported by null hypothesis Bayes factors. The 2^nd^-stage weighting parameter captures a mixture of model-based and model-free control. Model-free learning is captured via the learning rates at both stages as well as via the temporal difference (TD) lambda parameter, which reflects stage-skipping updates. Choice repetition is theoretically independent of model-free/model-based control but was empirically correlated with the model-based weight, possibly reflecting the degree of randomness of choices.

Next, we looked at the other model parameters to further test the hypothesis that spontaneous mind wandering impairs model-based but not model-free control (see Fig 3). First, we investigated the behavioral weight (inverse temperature parameter, *β*_*2*_) at the 2^nd^ task stage. Both model-based and model-free systems estimate the value (i.e., average long run reward) that each choice option is expected to deliver. The 2^nd^-stage weighting parameter simply captures how strongly reward learning influences 2^nd^-stage choices, and cannot distinguish whether this originates from the model-based or the model-free system, since both systems make the same predictions for behavior. Given that the model-based contribution should be impaired by spontaneous mind wandering (whereas the model-free contribution should be unaffected), we expected a decrease in *β*_*2*_. Indeed, as theoretically expected, the *β*_*2*_ parameter was correlated with the model-based weight (see S1 File). Moreover, consistent with our theoretical expectation, *β*_*2*_ was reduced with high levels of spontaneous mind wandering (continuous score: *b* = -0.22, *SE* = 0.082, *t* = -2.69, *p* = .012, *BF*_*10*_ = 13.7; median split: *b* = -0.50, *SE* = 0.178, *t* = -2.80, *p* = .009, *BF*_*10*_ = 11.0).

Additional model parameters estimate aspects of the model-free system, including *α*_*1*_, *α*_*2*_, representing learning rates, as well as *λ*, reflecting stage-skipping updates. If spontaneous mind wandering does not impair the functioning of the model-free system, then these model parameters should not be related to spontaneous mind wandering, which we indeed found in the data (for all three parameters in continuous and median-split analyses: *p*-values > .19 and null hypothesis Bayes factors 0.28 < *BF*_*10*_ < 1.06, suggesting that the data do not fully constrain the hypotheses).

Theoretically, the remaining model-parameter, the repetition factor, is neither linked to model-free nor to model-based control. However, we found a strong correlation between the repetition factor and the model-based weight, suggesting that both parameters may capture common variance, possibly a low influence of randomness in choices. We found that the repetition factor was reduced with increasing levels of mind wandering (continuous: *b* = -0.16, *SE* = 0.069, *t* = -2.26, *p* = 0.031, *BF*_*10*_ = 5.9; median-split: *b* = -0.39, *SE* = 0.15, *t* = -2.65, *p* = 0.013, *BF*_*10*_ = 9.1), possibly reflecting the factor that spontaneous mind wandering reduced model-based control and increased randomness of choices.

To summarize, we found effects of spontaneous mind wandering on the model-based weight, on the 2^nd^-stage weight, and on the repetition factor. Given that estimates of these three model parameters were highly correlated (see S1 Fig), effects of spontaneous mind wandering on the three parameters may capture the same model-based influence on choices, may not capture independent processes, and do surely not represent independent evidence. Importantly, they are all in line with the hypothesis that spontaneous mind wandering impairs model-based decision-making.

We also tested whether deliberate mind wandering affected any of the model parameters. However, we did not find a single significant effect (*p*-values > .26; uncorrected for multiple comparisons), neither for the continuous nor for the median-split analysis, consistent with our a priori expectation that deliberate mind wandering should not be prominent in the lab-environment in a demanding and difficult cognitive task like the two-step task.

## Discussion

In the current study, we used the two-step task to study how spontaneous mind wandering relates to model-based versus model-free decision making. As hypothesized, we found that spontaneous mind wandering was associated with reduced model-based control, but not with (reduced) model-free control.

Our results support the perceptual decoupling hypothesis that mind wandering interferes with task-related thoughts [14]. Moreover, the levels-of-inattention hypothesis [21] postulates that weak levels of mind wandering interfere only with high-level cognitive processes such as used in model-based decision making, but leaves lower-level cognitive processes–such as in model-free decision making–unaffected. Our results that spontaneous mind wandering relates to reduced higher-level model-based decision making, but not to lower-level model-free decision making, thus support the existence of such states of weak mind wandering.

Our results also inform theories of model-free versus model-based decision making. Given that mind wandering is thought to impair higher-level cognitive processing [10,16,21], our results add to previous reports that the model-based system relies on controlled processes including cognitive capacities such as working memory and processing speed [5,7,8,35], and thus depends on the allocation of attention to the external task environment. Indeed, we found the effect of spontaneous mind wandering on model-based control was nearly as strong as the strongest cognitive effect on model-based control we found before, i.e., processing speed [7]. By contrast, our current results suggest that model-free control seems to be relatively independent of attention being allocated to the external task environment [5], suggesting it is relatively independent of higher-level cognitive processing as theoretically expected.

Interestingly, mind wandering has recently been linked to model-based reasoning in a different way: evidence suggests model-based reasoning can take place outside of the original task environment [36,37], and is associated with default-mode activity [38] and spontaneous replay of new experiences [39,40], suggesting that the process of mind wandering may (partially) reflect model-based planning for other tasks or goals. This may also happen in the current task, where individuals with a strong tendency for spontaneous mind wandering may occupy their model-based decision making with planning for other (real-life) tasks, thus interfering with model-based planning during two-step task performance. The present results therefore suggest that mind wandering may reflect model-based offline replay for (real-life) tasks outside the current lab situation. Based on normative models of optimal replay [41], this perspective suggests that the brain may prioritize replay in an optimal way to maximize rewards, which–depending on their relative importance–may result in task-related attention or in task-unrelated planning for other goals reflecting mind wandering. This process may go awry in psychiatric diseases (e.g., depression): these might elicit mind wandering, which might provide a mechanism to reduce task-related model-based decision-making, leading to bad decisions and a vicious disease cycle.

An important limitation of the current study is, that we assessed mind wandering as an individual-difference variable and we can therefore not draw causal inferences concerning mind wandering causing reduced model-based control. Future studies are needed that utilize thought probes to assess individual mind wandering episodes during two-step task performance, and additional experimental manipulations of mind wandering frequency could also provide more clarity. As a further limitation, statistical power in the present study was only at 76%, and thus slightly below the recommended level of 80%. Again, future studies are thus needed to replicate the current findings. However, importantly, we found that the critical influence of spontaneous mind wandering on the model-based weight was supported also by a Bayes factor null hypothesis analysis, which is applicable also in situations where power is slightly reduced.

## Supporting information

S1 Fig Parameter recovery. Estimated (“recovery”) parameters per subject are plotted as a function of the true parameters used in the data simulation (the latter are the parameter estimates based on the empirical data). Parameters include the model-free weight (beta MF), the second-stage weight (beta 2), learning rates at 1^st^ stage (alpha 1) and at 2^nd^ stage (alpha 2), TD lambda (lambda), the model-based weight (beta MB), and the choice repetition parameter (rep).
(DOCX)

S2 Fig Correlations between estimates of all seven model parameters. *, *p* < .05; **, *p* < .01; ***, *p* < .001. Density plots are shown at the diagonal.
(DOCX)

S3 Fig Comparing the empirical data with model predictions using statistical markers of model-free and model-based learning. The main effect of reward, reflecting model-free control, as well as the interaction reward x transition frequency, reflecting model-based control, are shown for individuals with high versus low levels of spontaneous mind wandering (median split). Results are presented for the empirical data and for predictions from the fitted dual-control model. Error bars are S.E.M.
(DOCX)

S4 Fig Sensitivity analysis for the effect of spontaneous mind wandering on the model-based weight. The Bayes factor (BF10) is plotted as a function of the prior standard deviation of a normal distribution (defining the prior for the influence of spontaneous mind wandering on the model-based weight), which was truncated at zero to take only positive values, reflecting our a priori hypothesis that spontaneous mind wandering impaired model-based choice.
(DOCX)

S1 File (DOCX)
