# Correction: Spontaneous mind wandering impairs model-based decision making

**DOI:** 10.1371/journal.pone.0315190

**Published:** 2024-12-04

**Authors:** Shuyan Liu, Milena Rabovsky, Daniel J. Schad

The Supporting Information files are uploaded incorrectly. Please view the correct S1–S4 Figs and S1 File below.
