# Supplementary material for: Spontaneous mind wandering impairs model-based decision making
Source: PLoS One. 2023 Jan 26;18(1):e0279532. doi: 10.1371/journal.pone.0279532 (PMC9879536; doi:10.1371/journal.pone.0279532)
Supplement: S1 Fig — Estimated (“recovery”) parameters per subject are plotted as a function of the true parameters used in the data simulation (the latter are the parameter estimates based on the empirical data). Parameters include the model-free weight (beta MF), the second-stage weight (beta 2), learning rates at 1st stage (alpha 1) and at 2nd stage (alpha 2), TD lambda (lambda), the model-based weight (beta MB), and the choice repetition parameter (rep). (DOCX) [file pone.0279532.s001.docx]

$$P\left( a_{i,t}=a | s_{i,t} \right)=\frac{exp(Q_{net}\left( s_{i,t},a \right)+p\cdot rep(a))}{\sum_{a'} exp(Q_{net}\left( s_{i,t},a' \right)+p\cdot rep(a'))}$$

**

**Supplementary Fig 1**. Parameter recovery. Estimated (“recovery”) parameters per subject are plotted as a function of the true parameters used in the data simulation (the latter are the parameter estimates based on the empirical data). Parameters include the model-free weight (beta MF), the second-stage weight (beta 2), learning rates at 1^st^ stage (alpha 1) and at 2^nd^ stage (alpha 2), TD lambda (lambda), the model-based weight (beta MB), and the choice repetition parameter (rep).
