# Supplementary material for: Spontaneous mind wandering impairs model-based decision making
Source: PLoS One. 2023 Jan 26;18(1):e0279532. doi: 10.1371/journal.pone.0279532 (PMC9879536; doi:10.1371/journal.pone.0279532)
Supplement: S2 Fig — *, p < .05; **, p < .01; ***, p < .001. Density plots are shown at the diagonal. (DOCX) [file pone.0279532.s002.docx]

$$P\left( a_{i,t}=a | s_{i,t} \right)=\frac{exp(Q_{net}\left( s_{i,t},a \right)+p\cdot rep(a))}{\sum_{a'} exp(Q_{net}\left( s_{i,t},a' \right)+p\cdot rep(a'))}$$

**

**Supplementary Fig 2.** Correlations between estimates of all seven model parameters. *, *p* < .05; **, *p* < .01; ***, *p* < .001. Density plots are shown at the diagonal.
