# Supplementary material for: Spontaneous mind wandering impairs model-based decision making
Source: PLoS One. 2023 Jan 26;18(1):e0279532. doi: 10.1371/journal.pone.0279532 (PMC9879536; doi:10.1371/journal.pone.0279532)
Supplement: S3 Fig — The main effect of reward, reflecting model-free control, as well as the interaction reward x transition frequency, reflecting model-based control, are shown for individuals with high versus low levels of spontaneous mind wandering (median split). Results are presented for the empirical data and for predictions from the fitted dual-control model. Error bars are S.E.M. (DOCX) [file pone.0279532.s003.docx]

$$P\left( a_{i,t}=a | s_{i,t} \right)=\frac{exp(Q_{net}\left( s_{i,t},a \right)+p\cdot rep(a))}{\sum_{a'} exp(Q_{net}\left( s_{i,t},a' \right)+p\cdot rep(a'))}$$


**Supplementary Fig 3.** Comparing the empirical data with model predictions using statistical markers of model-free and model-based learning. The main effect of reward, reflecting model-free control, as well as the interaction reward x transition frequency, reflecting model-based control, are shown for individuals with high versus low levels of spontaneous mind wandering (median split). Results are presented for the empirical data and for predictions from the fitted dual-control model. Error bars are S.E.M.
