# Supplementary material for: Spontaneous mind wandering impairs model-based decision making
Source: PLoS One. 2023 Jan 26;18(1):e0279532. doi: 10.1371/journal.pone.0279532 (PMC9879536; doi:10.1371/journal.pone.0279532)
Supplement: S4 Fig — The Bayes factor (BF10) is plotted as a function of the prior standard deviation of a normal distribution (defining the prior for the influence of spontaneous mind wandering on the model-based weight), which was truncated at zero to take only positive values, reflecting our a priori hypothesis that spontaneous mind wandering impaired model-based choice. (DOCX) [file pone.0279532.s004.docx]

$$P\left( a_{i,t}=a | s_{i,t} \right)=\frac{exp(Q_{net}\left( s_{i,t},a \right)+p\cdot rep(a))}{\sum_{a'} exp(Q_{net}\left( s_{i,t},a' \right)+p\cdot rep(a'))}$$


**Supplementary Fig 4.** Sensitivity analysis for the effect of spontaneous mind wandering on the model-based weight. The Bayes factor (BF10) is plotted as a function of the prior standard deviation of a normal distribution (defining the prior for the influence of spontaneous mind wandering on the model-based weight), which was truncated at zero to take only positive values, reflecting our a priori hypothesis that spontaneous mind wandering impaired model-based choice.
