# Supplementary material for: Spontaneous mind wandering impairs model-based decision making
Source: PLoS One. 2023 Jan 26;18(1):e0279532. doi: 10.1371/journal.pone.0279532 (PMC9879536; doi:10.1371/journal.pone.0279532)
Supplement: S1 File — (DOCX) [file pone.0279532.s005.docx]

**Supplementary Information**

Model description

We used the computational dual-control model by [[3](#_ENREF_3)] (For a full model description see their supplementary material as well as [[7](#_ENREF_7)]), and used the re-parametrization introduced by [[8](#_ENREF_8)]. The model assumes that two different learning systems are in place in parallel: a model-free system is thought to be grounded in the brain’s dopaminergic system [[1](#_ENREF_1)], and learns backwards from experience of prediction errors, and a model-based system anticipates futures consequences of actions based on a cognitive map of the (task) environment.

Each of the two systems learns to perform actions to gain rewards on the task. The task consists of three states (one first-stage state: s_A_; and two second-stage states: s_B_ and s_C_). In each state, the subject can take one of two actions (a_A_ and a_B_). A first-stage action leads to probabilistic transitions to either s_B_ (70% of trials) or to s_C_ (30%), and the other first-stage action has reversed transition probabilities. Both the model-based and the model-free subcomponents of the model aim to learn a state-action value function Q(s, a) mapping each state-action pair to its expected future value, that is, each system learns to predict how much reward is obtained by taking a certain action. On trial *t*, we denoted the first-stage state (always s_A_) by s_1,t_, the second-stage state by s_2,t_, the first- and second-stage actions by a_1,t_ and a_2,t_ , and the first- and second-stage rewards as r_1,t_ (always zero) and r_2,t_.

The model-free system learns using temporal difference (TD) learning, i.e., based on prediction errors, which are computed once an action is taken and the consequences are experienced.

𝛿i,t = 𝑟i,t + 𝑄TD(𝑠i+1,t, 𝑎i+1,t) – 𝑄TD(𝑠i,t, 𝑎i,t)

Here, *i* denotes the state (at first stage, *i* = 1; second stage, *i* = 2; final stage, *i* = 3) and *r* denotes reward in state *i* and trial *t*. Prediction errors are then used to update action values:

𝑄TD(𝑠i,t, 𝑎i,t) = 𝑄TD(𝑠i,t, 𝑎i,t) + 𝛼i𝛿i,t

Moreover, prediction errors at the second-stage action are used to inform first-stage action values using eligibility parameter 𝜆 via:

𝑄TD(𝑠1,t , 𝑎1,t ) = 𝑄TD (𝑠1,t , 𝑎1,t ) + 𝛼i 𝜆𝛿2,t

The model-based system anticipates future consequences of actions by considering possible transitions according to their probabilities. We characterized model-based transition learning by assuming participants count how many transitions they have observed, and then assuming they simply choose between the two possibilities: P( s_B_|s_A_, α_A_ ) = 0.7, P( s_c_|s_A_, α_B_) = 0.7, or, vice versa P( s_B_|s_A_, α_A_ ) = 0.3, P( s_C_|s_A_, α_B_ ) = 0.3. In the model-based system, to compute the (Q-)value of a given action, 𝑄MB(𝑠A, 𝑎j), each possible transition is considered separately, i.e., the transition to s_B_ and to s_C_. For each transition, the transition probability (0.3 or 0.7) is multiplied with the value of the highest-value action at that successor state. These products (probability x value) are then summed across both possible second-stage states to compute 𝑄MB(𝑠A, 𝑎j). At the second-stage, the problem of learning immediate rewards is equivalent to that for temporal-difference (TD) learning above, since Q_TD_(s_2,t_,a_2,t_) is just an estimate of the immediate reward r_2,t_; with no further steps to anticipate.

The value predictions of the model-based and model-free systems are combined according to:

𝑄net(𝑠A, 𝑎j) = $\beta_{MB}$𝑄MB(𝑠A, 𝑎j) + $\beta_{MF}$𝑄TD(𝑠A, 𝑎j)

where $\beta_{MB}$and $\beta_{MF}$ are weighting parameters that determine how strongly each of the two systems impacts on choices.

The probability to choose a given first-stage action is computed via the softmax rule:

$$P\left( a_{i,t}=a | s_{i,t} \right)=\frac{\exp(Q_{net}\left( s_{i,t},a \right)+p\cdot rep(a))}{\sum_{a'} \exp(Q_{net}\left( s_{i,t},a' \right)+p\cdot rep(a'))}$$

The parameter p captures an overall probability to repeat the action from the last trial.

Consistency checks of model fit

To study model fit more closely, and to compare model behavior to human behavior, we simulated data based on the estimated model parameters. **Supplementary Fig 3** shows the main effect of reward, reflecting model-free control, as well as the interaction of reward x transition probability, reflecting model-based control. This was computed using the empirical data (“Data”) as well as the computational model (“Model”). The results show that the reward x transition interaction (model-based control) from the empirical data was well captured by the computational model: the model roughly captured the average size of the interaction. Moreover, in the empirical data the interaction was reduced in subjects with high mind wandering, and this was also visible in the computational model. Next, the main effect of reward, i.e., model-free control, which was present in the empirical data, was mostly absent in the computational model, suggesting the model under-estimated model-free control of behavior. However, in the empirical data the main effect of reward did not differ between high versus low mind wandering, and this was also the case in the computational model, suggesting the model captured this absence of individual differences well.
