# Supplementary figures and images for: Correction: Spontaneous mind wandering impairs model-based decision making
Source: PLoS One. 2024 Dec 4;19(12):e0315190. doi: 10.1371/journal.pone.0315190 (PMC11616804; doi:10.1371/journal.pone.0315190)

**beta MF:  $r = 0.92$** 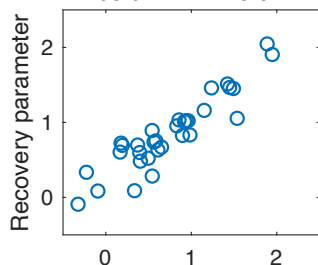**beta 2:  $r = 0.96$** 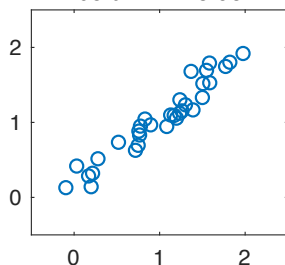**alpha 1:  $r = 0.84$** 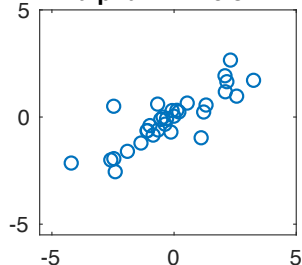**alpha 2:  $r = 0.89$** 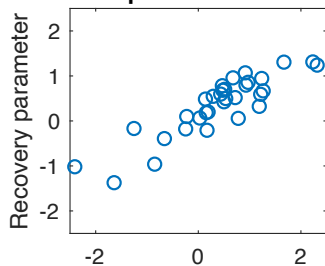**lambda:  $r = 0.84$** 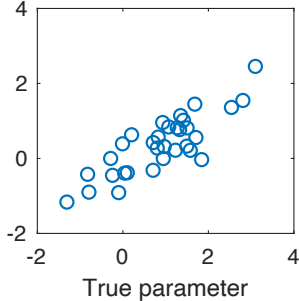**beta MB:  $r = 0.88$** 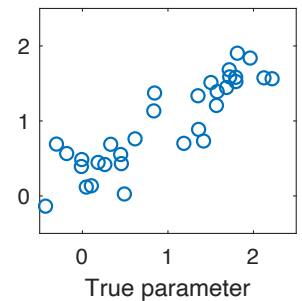**rep:  $r = 0.95$** 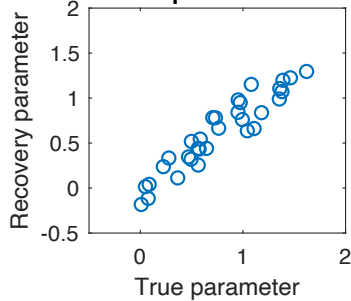

Supplement: S1 Fig — (PDF) [file pone.0315190.s001.pdf]

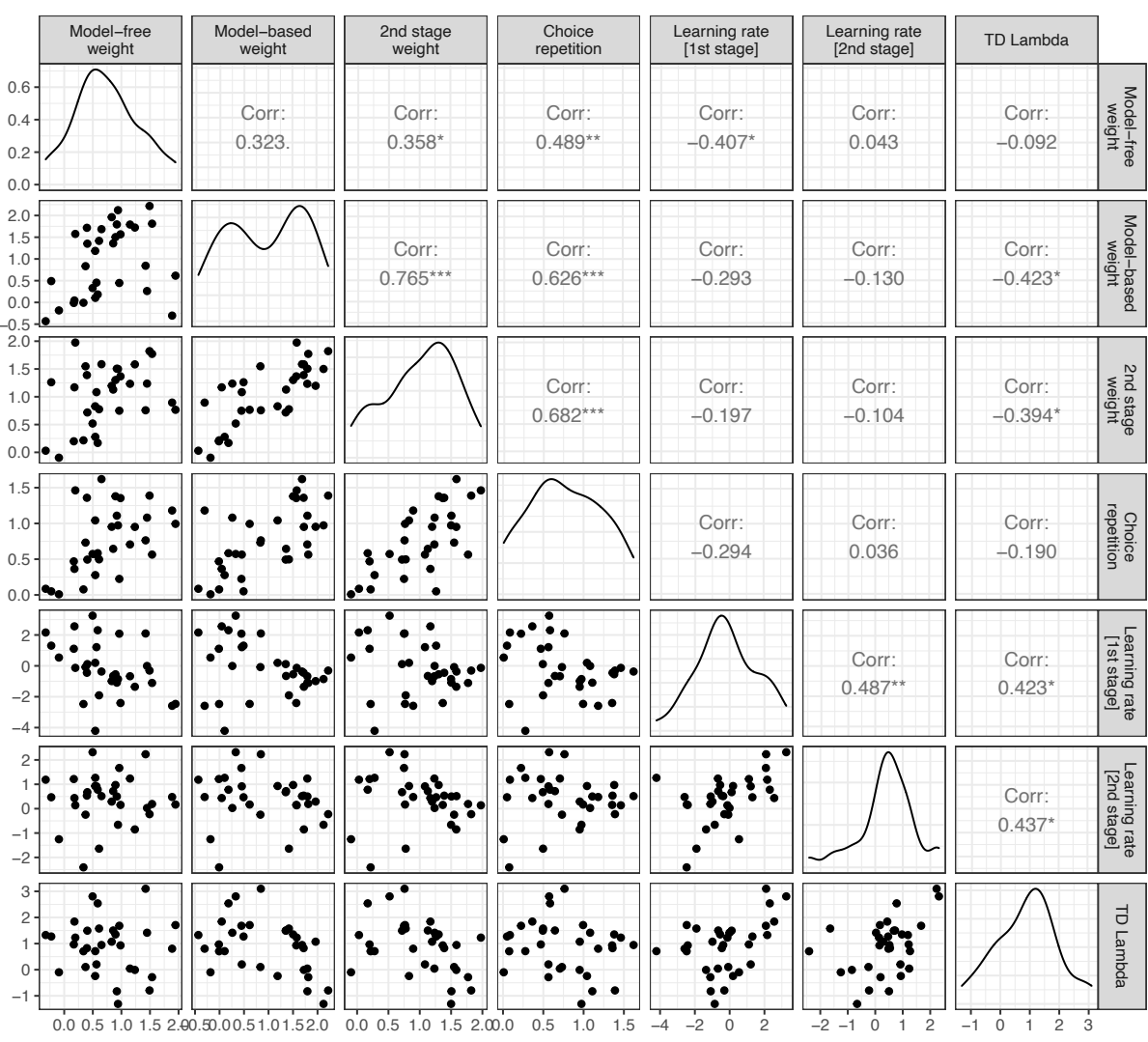

Supplement: S2 Fig — (PDF) [file pone.0315190.s002.pdf]

Estimate

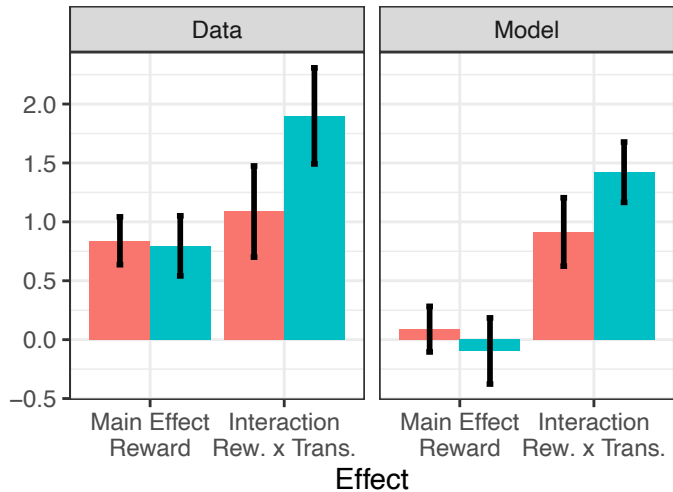

Supplement: S3 Fig — (PDF) [file pone.0315190.s003.pdf]

Bayes factor [BF10]

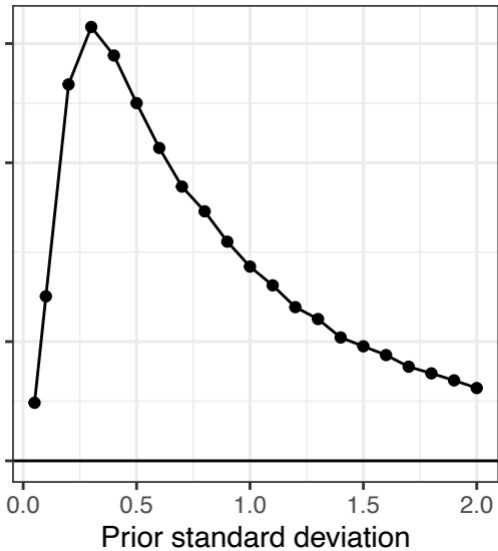

Supplement: S4 Fig — (PDF) [file pone.0315190.s004.pdf]
