# Supplementary material for: Correction: Spontaneous mind wandering impairs model-based decision making
Source: PLoS One. 2024 Dec 4;19(12):e0315190. doi: 10.1371/journal.pone.0315190 (PMC11616804; doi:10.1371/journal.pone.0315190)
Supplement: S1 File — (PDF) [file pone.0315190.s005.pdf]

Supplementary Information

Model description

We used the computational dual-control model by [3] (For a full model description see their supplementary material as well as [7]), and used the re-parametrization introduced by [8]. The model assumes that two different learning systems are in place in parallel: a model-free system is thought to be grounded in the brain's dopaminergic system [1], and learns backwards from experience of prediction errors, and a model-based system anticipates futures consequences of actions based on a cognitive map of the (task) environment.

Each of the two systems learns to perform actions to gain rewards on the task. The task consists of three states (one first-stage state: s_A; and two second-stage states: s_B and s_C). In each state, the subject can take one of two actions (a_A and a_B). A first-stage action leads to probabilistic transitions to either s_B (70% of trials) or to s_C (30%), and the other first-stage action has reversed transition probabilities. Both the model-based and the model-free subcomponents of the model aim to learn a state-action value function Q(s, a) mapping each state-action pair to its expected future value, that is, each system learns to predict how much reward is obtained by taking a certain action. On trial *t*, we denoted the first-stage state (always s_A) by $s_{1,t}$, the second-stage state by $s_{2,t}$, the first- and second-stage actions by $a_{1,t}$ and $a_{2,t}$, and the first- and second-stage rewards as $r_{1,t}$ (always zero) and $r_{2,t}$.

The model-free system learns using temporal difference (TD) learning, i.e., based on

prediction errors, which are computed once an action is taken and the consequences are experienced.

$$\delta_{i,t} = r_{i,t} + Q_{TD}(s_{i+1,t}, a_{i+1,t}) - Q_{TD}(s_{i,t}, a_{i,t})$$

Here, i denotes the state (at first stage, i = 1; second stage, i = 2; final stage, i = 3) and r denotes reward in state i and trial t. Prediction errors are then used to update action values:

$$Q_{TD}(s_{i,t}, a_{i,t}) = Q_{TD}(s_{i,t}, a_{i,t}) + \alpha_i \delta_{i,t}$$

Moreover, prediction errors at the second-stage action are used to inform first-stage action values using eligibility parameter λ via:

$$Q_{TD}(s_{1,t}, a_{1,t}) = Q_{TD}(s_{1,t}, a_{1,t}) + \alpha_i \lambda \delta_{2,t}$$

The model-based system anticipates future consequences of actions by considering possible transitions according to their probabilities. We characterized model-based transition learning by assuming participants count how many transitions they have observed, and then assuming they simply choose between the two possibilities: P($s_B|s_A$, α_A) = 0.7, P($s_c|s_A$, α_B) = 0.7, or, vice versa P($s_B|s_A$, α_A) = 0.3, P($s_C|s_A$, α_B) = 0.3. In the model-based system, to compute the (Q-)value of a given action, $Q_{MB}(s_A, a_j)$, each possible transition is considered separately, i.e., the transition to

s_B and to s_C. For each transition, the transition probability (0.3 or 0.7) is multiplied with the value of the highest-value action at that successor state. These products (probability x value) are then summed across both possible second-stage states to compute $Q_{MB}(s_A, a_j)$. At the second-stage, the problem of learning immediate rewards is equivalent to that for temporal-difference (TD) learning above, since $Q_{TD}(s_{2,t},a_{2,t})$ is just an estimate of the immediate reward $r_{2,t}$; with no further steps to anticipate.

The value predictions of the model-based and model-free systems are combined according to:

$$Q_{net}(s_A, a_j) = \beta_{MB}\, Q_{MB}(s_A, a_j) + \beta_{MF}\, Q_{TD}(s_A, a_j)$$

where β_{MB} and β_{MF} are weighting parameters that determine how strongly each of the two systems impacts on choices.

The probability to choose a given first-stage action is computed via the softmax rule:

$$P\left(a_{i,t} = a \middle| s_{i,t}\right) = \frac{\exp(Q_{net}\left(s_{i,t}, a\right) + p \cdot rep(a))}{\sum_{a'} \exp(Q_{net}\left(s_{i,t}, a'\right) + p \cdot rep(a'))}$$

The parameter p captures an overall probability to repeat the action from the last trial.

Consistency checks of model fit

To study model fit more closely, and to compare model behavior to human behavior, we simulated data based on the estimated model parameters. **Supplementary Fig 3** shows the main effect of reward, reflecting model-free control, as well as the interaction of reward x transition probability, reflecting model-based control. This was computed using the empirical data ("Data") as well as the computational model ("Model"). The results show that the reward x transition interaction (model-based control) from the empirical data was well captured by the computational model: the model roughly captured the average size of the interaction. Moreover, in the empirical data the interaction was reduced in subjects with high mind wandering, and this was also visible in the computational model. Next, the main effect of reward, i.e., model-free control, which was present in the empirical data, was mostly absent in the computational model, suggesting the model under-estimated model-free control of behavior. However, in the empirical data the main effect of reward did not differ between high versus low mind wandering, and this was also the case in the computational model, suggesting the model captured this absence of individual differences well.
